# Post-glacial biogeography of trembling aspen inferred from habitat models and genetic variance in quantitative traits

**DOI:** 10.1038/s41598-017-04871-7

**Published:** 2017-07-05

**Authors:** Chen Ding, Stefan G. Schreiber, David R. Roberts, Andreas Hamann, Jean S. Brouard

**Affiliations:** 1 University of Alberta, Department of Renewable Resources, 751 General Services Building Edmonton, AB, Alberta, T6G 2H1 Canada; 2 Isabella Point Forestry Ltd., 331 Roland Road, Salt Spring Island, BC, Alberta, V8K 1V1 Canada

## Abstract

Using species distribution models and information on genetic structure and within-population variance observed in a series of common garden trials, we reconstructed a historical biogeography of trembling aspen in North America. We used an ensemble classifier modelling approach (RandomForest) to reconstruct palaeoclimatic habitat for the periods 21,000, 14,000, 11,000 and 6,000 years before present. Genetic structure and diversity in quantitative traits was evaluated in common garden trials with 43 aspen collections ranging from Minnesota to northern British Columbia. Our main goals were to examine potential recolonisation routes for aspen from southwestern, eastern and Beringian glacial refugia. We further examined if any refugium had stable habitat conditions where aspen clones may have survived multiple glaciations. Our palaeoclimatic habitat reconstructions indicate that aspen may have recolonised boreal Canada and Alaska from refugia in the eastern United States, with separate southwestern refugia for the Rocky Mountain regions. This is further supported by a southeast to northwest gradient of decreasing genetic variance in quantitative traits, a likely result of repeated founder effects. Stable habitat where aspen clones may have survived multiple glaciations was predicted in Mexico and the eastern United States, but not in the west where some of the largest aspen clones have been documented.

## Introduction

Trembling aspen (*Populus tremuloides* Michx.) is the most frequent and genetically diverse forest tree in North America, occupying many ecological site types from Mexico to Alaska in the west, and across Canada and the United States to the Atlantic ocean in the east^1–3^. Aspen is capable of colonizing newly available habitat, yet differs from typical pioneer species in that it can persist in colonised environments for thousands of years through clonal reproduction. Due to its life history, large range, and wide ecological amplitude, aspen is an interesting organism to address questions concerning ecological genetics, physiology, and biogeography.

Aspen can colonise marginal habitat and survive disturbance events by root suckering^4^. Seed production is commonly observed but seedling establishment is less common than suckering, especially in the semiarid areas of western North America^2, 5^. In moister habitat of the northern Rocky Mountains and eastern North America, seedling establishment occurs more frequently^5, 6^. Once an individual is established, it will send out lateral roots from which hundreds of ramets can originate. The clone increases in size as each ramet also contributes distally to the expanding root system from which new stems can be formed^4^. The largest confirmed aspen clone to date (known as “Pando”) covers 43 ha and comprises 47,000 stems with an estimated biomass of 6,000 t^5, 7, 8^. In the eastern United States, the average clone size has been estimated to be approximately 0.04 ha, with exceptional individuals reaching 14 ha^4, 9, 10^. In central Canada, average clone sizes were reported around 0.08 ha, with the largest clones reaching 1.5 ha^11^.

Several genetic studies have also shown that trembling aspen shows exceptionally high levels of genetic diversity, but little among-population genetic differentiation in neutral genetic markers, such as isozymes, microsatellites or other molecular markers^7, 12–21^. The highest levels of genetic diversity with an expected heterozygosity (H_e_) of 0.42 were reported for Alberta by^12^, but other studies in the region showed more typical levels of genetic diversity for the species with an H_e_ of 0.29^22^. Electrophoretic surveys of aspen in eastern populations (e.g. Minnesota and Ontario) were lower with H_e_ rates of 0.22 and 0.25, respectively^15, 16^.

In a recent range-wide study of genetic structure and diversity based on microsatellite markers, Callahan, *et al*.^20^ identified a pronounced geographic differentiation into a genetically more diverse northern cluster (Alaska, Canada, northeastern US) and a slightly less diverse southwestern cluster (western US and Mexico) while showing no evidence of higher genetic diversity in Alberta. However, due to different rates and mechanisms of mutations in isozyme versus microsatellite marker systems the outlined results may not be contradictory^14, 23^.

A different approach to investigate genetic structure and diversity is to assess genetic differences of quantitative traits in common garden experiments. Such data usually does not provide insight into the biogeographic history of a species, because the traits evolve too quickly in response to current environments. In the case of aspen, however, where clones may have persisted for thousands of years, genetic variance in quantitative traits or adaptational lag may provide additional clues regarding the migration history of the species. In a reciprocal transplant experiment, Schreiber, *et al*.^24^ showed evidence for strong suboptimality in adaptive traits, and suboptimality in quantitative traits could potentially be explained by considering aspen’s clonal life history, with populations being adapted to fossil climate conditions^25^.

In fact, it has been speculated that aspen clones may be millions of years old and have survived dozens or hundreds of glacial cycles^2, 4, 5^. Although precise dating of aspen clones remains an elusive task, recent studies have drawn some boundaries. Ally, *et al*.^26^ found that the upper boundary for the age of aspen clones at two study sites in British Columbia is approximately 4,000 and 10,000 years, which corresponds well with the timing of glacial retreat at those two sites. Relating clone size with age, however, proved not possible. Speculations about very large clones that may have persisted through repeated glacial cycles are also not supported by Mock, *et al*.^17^, who studied the largest known clone “Pando” and concluded that it has a low frequency of somatic mutations at microsatellite loci and is not likely to be more than several thousand years old.

Another valuable approach to address questions concerning biogeography and species migration are species distribution models. These models use observed species range data in combination with environmental predictors (typically climate) to generate statistical relationships, which can be used to project probabilities of species presence from new environmental data^27^. Although more typically used as a risk assessment tool for future climate change e.g. ref. 28, they are also employed to reconstruct biogeographical histories of species e.g. refs 29–33.

In this study, we contribute reconstructions of glacial refugia and post-glacial migration histories for aspen by means of species distribution models. Our primary goal is to use habitat reconstructions to augment inferences of putative glacial refugia that are based on geographic patterns in neutral genetic markers. Callahan *et al*.^20^’s more diverse northern cluster and a less diverse southwestern cluster suggests two refugia for the species south of the ice sheet, but others have proposed that boreal species may also had refugia in ice-free Beringia, allowing for southward post-glacial recolonisation routes^34, 35^. Secondly, we investigate whether habitat reconstructions support the possibility of very old clones that may have persisted through multiple glaciations by climate conditions staying within their environmental tolerances. Last, we contribute an analysis of genetic diversity and adaptational lag in quantitative traits based on field trials. Since aspen clones may have persisted for thousands of years in many current locations, their adaptational lag would provide additional insight as to what climate conditions they have experienced in the past and what migration paths would be consistent with observed lags and gradients in genetic diversity.

## Results

### Genetic differentiation and adaptation

Because aspen clones may have persisted for thousands of years, any adaptational lag relative to current environments may provide additional clues as to what climate conditions they have experienced in the past and what migration direction would be consistent with the observed lag.

**Figure 1:**
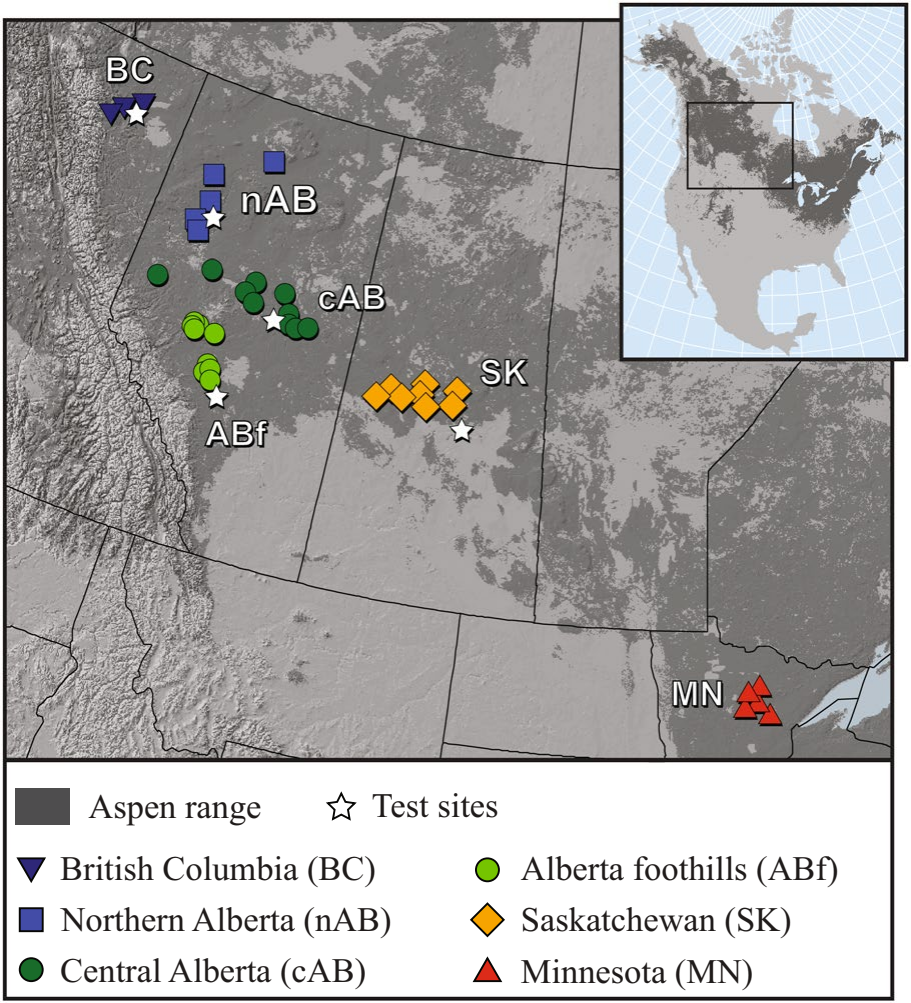
Collection locations and test sites of the aspen provenance trial series that was used to quantify within-population genetic diversity and adaptational lag of aspen populations. The map was created with ArcGIS v9.3 (http://esri.com).

**Figure 2:**
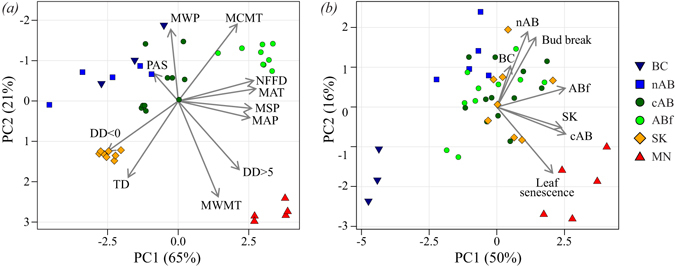
Principal components analyses for climate conditions at seed source locations (**a**) and multi-trait measurements in common garden trials (**b**). Vector labels represent the input variables. Symbols in (**a**) represent the geographic location of provenances; symbols in (**b**) represent the provenance collections. Vector labels in (**a**): PAS = precipitation as snow (mm), MWP = mean winter precipitation (°C), MCMT = mean coldest month temperature (°C), NFFD = number of frost free days, MAT = mean annual temperature (°C), MSP = mean summer precipitation (mm), MAP = mean annual precipitation (mm), DD > 5 = degree-days above 5 °C (growing degree-days), MWMT = mean warmest month temperature (°C), TD = temperature difference between MCMT and MWMT (or continentality, °C), DD < 0 = degree-days below 0 °C (chilling degree-days); Vector labels in (**b**): BC = height at British Columbia test site, nAB = height at northern Alberta test site, ABf = height at Alberta Foothills test site, SK = height at Saskatchewan test site, cAB = height at central Alberta test site, Bud break = timing of bud break at central Alberta test site, Leaf senescence = timing of leaf senescence at central Alberta test site.

To concisely summarize multi-trait measurements at five test sites as well as climate conditions at seed source locations (Fig. 1), we use a principal component analysis (Fig. 2). The vectors represent components loadings, which are the correlations of the principal components with the original variables. The strength of the correlation is indicated by the vector length, and the direction indicates which seed sources have high values for the original variables. Climate conditions of seed source locations show a number of distinct groups (Fig. 2a). Minnesota sample site climates are characterised by warm and long summers (MWMT, DD > 5), Saskatchewan sources have the longest and harshest winter conditions (DD < 0, opposite MCMT), the Alberta Foothills sources have the strongest maritime influence with mild winters (MCMT, opposite DD < 0 and TD) and high precipitation (MAT, MSP), whereas the boreal forest locations (cAB, nAB, BC) are characterised by cool summers and short growing seasons, as well as dry growing season conditions (opposite MWMT, MAP, DD > 5).

Regarding genetic structure of populations (Fig. 2b), only two groups of samples are clearly differentiated from the other groups based on the measured traits. In this figure, symbols represent provenance collections, and vectors represent height measurements at five test sites (arrow labels BC, nAB, cAB, ABf, SK), plus bud break and leaf senescence measurements at one site (cAB). Provenances from British Columbia (BC) are characterised by poor relative performance at most test sites, particularly under the mild climates of the Alberta Foothills test site (opposite to most height vectors, particularly ABf). BC provenances are also characterised by early bud break. The other group that is clearly separated comprises the Minnesota (MN) provenances, which grow well at most test sites, particularly the central Alberta site (cAB). They are also characterised by late leaf senescence. The remaining groups of samples are not genetically differentiated in the measured traits, although the climate conditions of their origins is quite distinct, particularly for the Saskatchewan (SK) and Alberta Foothills (ABf) source climates (*cf*. Fig. 2a).

### Regional within-population genetic variation

Residual variance components of growth and adaptive traits by region of origin reveal the Alberta Foothills and Minnesota as the most genetically diverse regions in quantitative traits (Table 1). If we ignore the sub-boreal Foothills location, a trend toward decreasing genetic diversity across aspen’s main boreal distribution from southeastern Minnesota to northwestern British Columbia is apparent in all measured traits (*cf*. Fig. 1). The gradient is most pronounced for height measurements (0.94 in MN to 0.61 in BC), and height measurements also have the highest accuracy of within-population diversity estimates, because they were evaluated at five sites. With respect to timing of bud break, all western Canadian provenances are fairly homogenous only contrasting with the Minnesota provenances with much higher within-population diversity. The Alberta Foothills region has the highest residual variation for the timing of leaf senescence followed by Minnesota.

**Table 1:** Measured adaptive traits and residual variance components summarised by region.

Region	Within-population variance components		
	Height	Bud break	Leaf senescence
BC Northeast	0.61 (0.06)	8.4 (1.7)	5.2 (2.7)
Northern AB	0.71 (0.04)	8.9 (1.3)	6.6 (1.4)
AB Foothills	0.87 (0.03)	9.2 (1.0)	10.3 (1.4)
Central AB	0.81 (0.03)	8.8 (0.8)	7.5 (0.8)
Saskatchewan	0.80 (0.04)	8.9 (0.9)	6.2 (0.7)
Minnesota	0.94 (0.06)	13.1 (1.5)	8.3 (1.2)

Standard errors (SE) in parentheses. Height measurements were taken at all five test sites. Bud break and leaf senescence was measured only at the central Alberta test site.

### Palaeoclimatic habitat reconstructions

While an out-of-bag validation indicated excellent model fit to modern plot data with an AUC of 0.91, a model validation against pollen and fossil data yielded an AUC of only 0.67. Although truly independent model validation statistics are always much lower than out-of-bag validations e.g. refs 36 and 37, the model used in this study fits fossil pollen data for aspen poorly. High error rates for fossil data are expected due to model limitations, inaccurate palaeoclimate reconstructions, but also because of the nature of the palaeoecological validation data itself. For example, pollen deposits are restricted to certain landscape features and topographic positions, such as bogs or lakes, where the sources of pollen are different from ecological habitats in the broader surroundings, leading to false positive sediment records. Particularly low AUC values for aspen compared to other western North American tree species were previously observed^38, 39^. The reason may be that pollen identification for poplar is difficult beyond the genus level and that poplar pollen is also fragile and prone to disintegration, which may also lead to false negatives in sediment records^40^.

**Figure 3:**
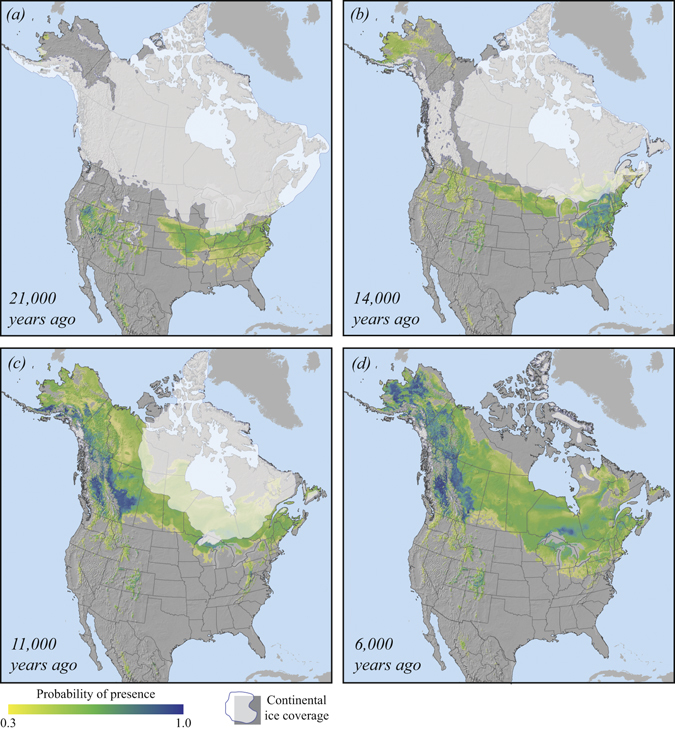
Palaeoclimatic habitat projections for trembling aspen based on the CCM1 general circulation model for (**a**) 21,000 years before present, (**b**) 14,000 years before present, (**c**) 11,000 years before present and (**d**) 6,000 years before present. The maps were created with ArcGIS v9.3 (http://esri.com).

Our historical projections of aspen habitat for 6,000, 11,000, 14,000 and 21,000 years before present (BP) show three potential glacial refugia in which aspen may have found suitable habitat during the last glacial maximum (Fig. 3). The predicted 21,000 years BP refugia are found in present-day Alaska, although small and with a low probability of presence, and in the southwestern and eastern United States (Fig. 3a). The maps highlight a potential contact zone located in the prairie provinces of western Canada in which populations from these three refugia may have merged after the retreat of the Wisconsin glaciers at around 11,000 years BP (Fig. 3c). The largest glacial refugium was predicted in the eastern United States, which may have contributed the highest genetic influx during recolonisation of the North American continent.

**Figure 4:**
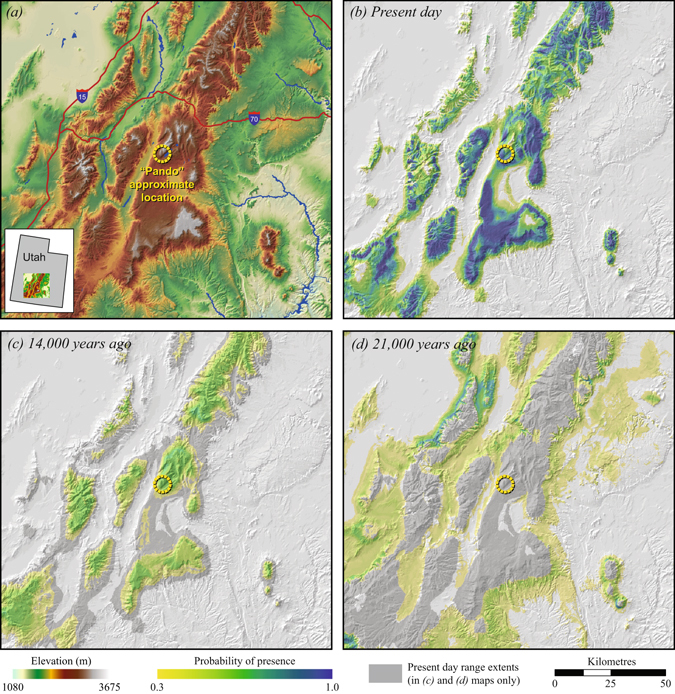
(**a**) Topographic map of south-central Utah highlighting the approximate location of the aspen clone “Pando”. Palaeoclimatic habitat projections for trembling aspen in south-central Utah (**b**) present day, (**c**) 14,000 years before present, (**d**) 21,000 years before present. The maps were created with ArcGIS v9.3 (http://esri.com).

**Figure 5:**
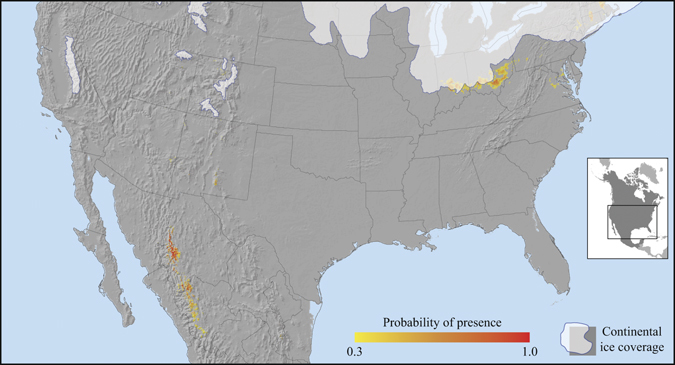
Probabilities of geographic locations in which aspen clones may have persisted through multiple glaciations. Data points were derived by multiplying the probability of presence estimates of the 1961–1990 reference climate with the 21,000 years before present period. The map was created with ArcGIS v9.3 (http://esri.com).

Figure 4 shows a higher resolution image of the same projections of aspen habitat for the Fish Lake National Forest in south central Utah, where the largest confirmed aspen clone “Pando” has been documented. The model predicts suitable habitat to emerge at the earliest at 14,000 years BP, and no suitable habitat is predicted in the vicinity of today’s location of the clone at the last glacial maximum at 21,000 years BP. At a larger scale, the overlap of suitable aspen habitat between the present and the last glacial maximum was obtained by multiplying probabilities of presence between the model outputs for the 1961–1990 baseline period and for 21,000 years BP (Fig. 5). The analysis reveals only a few locations in which aspen populations had a moderate or high probability of surviving multiple glaciations. These areas are located in eastern United States (southeastern Ohio) and the Sierra Madre mountain range in northeastern Mexico.

## Discussion

Palaeoclimatic habitat reconstructions suggest three potential glacial refugia for trembling aspen from which recolonisation may have occurred. The eastern United States represents the largest refugium with the highest probabilities of presence, followed by the low elevation areas of the southwestern United States, and Alaska. Although the modelled Alaska refugium was very small with a low-probability of presence, the possibility of aspen recolonisation from the north should not be excluded based on habitat reconstructions alone. This leaves three conceivable recolonisation scenarios for aspen: (Scenario 1) recolonisation of the boreal north almost exclusively from the southeast to northwest up into Alaska; (Scenario 2) recolonisation predominantly from the east but with contributions from either the southwestern or Alaskan refugia, and (Scenario 3) simultaneous recolonisation from all three glacial refugia with a contact zone in Alberta, Canada, potentially explaining high levels of genetic diversity documented by one study for this region.

Southwestern coastal and interior refugia are well documented for many western North American species e.g. reviewed by^41, 42^, and many interior plant species show genetic clusters that can be attributed to a further sub-structure of southwestern refugia. For example, Godbout *et al*.^43^ propose two well separated refugia in the Columbia River basin and the eastern Rocky Mountains to explain genetic structure within the interior variety of *Pinus contorta*. Northeastern refugia, just south of the ice sheet have also been well documented for several boreal tree species reviewed by^42^, implying either exclusive northwestern recolonisation paths (e.g. *Pinus banksiana*), or recolonisation from both the southwest and east (e.g. *Picea mariana*). In addition, there is evidence from both genetic data and fossil pollen records that several boreal species may also have found refuge in ice-free Beringia, allowing for southward post-glacial recolonisation routes^34, 35^. For aspen, two main genetic clusters in today’s populations have been identified: a northern group comprised of the Alaskan, Canadian and eastern United States populations, and a second group of southern Rocky Mountain populations presumably originating from a separate southwestern refugium^20^.

### Recolonisation from southwestern and eastern refugia

Our habitat reconstructions conform well to the genetic clusters described by Callahan, *et al*.^20^. In fact, they also confirm that an observed outlier in the southern cluster (a Yellowstone population), which according to the microsatellite data grouped into the northern cluster could have plausibly been recolonised from the east. Although westward extending habitat from eastern refugia at 14,000 years BP did not reach all the way to the Yellowstone region, it does extend well into Montana (Fig. 3b). It seems therefore likely that eastern population stretched all the way to the Rocky Mountain foothills at some point in time between 11,000 and 14,000 years BP, providing a complete southern front along the entire length of the Laurentian ice sheet. From here, northward recolonisation of boreal Canada and Alaska could have proceeded with little opportunity for genetic contributions from southwestern refugia. This provides an explanation for the large northern genetic cluster described by Callahan *et al*.^20^.

The hypothesis of northern recolonisation after the retreat of the ice sheet from the east (Scenario 1) is further supported by our finding of a southeast to northwest gradient of decreasing genetic variance in quantitative traits (Table 1). Such a gradient would be expected because of repeated founder effects during post-glacial migration westwards and northwards^44^. Patterns of adaptational lag in quantitative traits also fit well with this migration history. Aspen provenances from northeastern British Columbia are the least well-adapted populations in terms of growth, survival, phenology and frost hardiness compared to populations from Alberta and Minnesota^24^. Decreasing genetic diversity in combination with aspen’s clonal life history slow the process of adaptation to new environmental conditions, with current populations essentially being adapted to fossil climates^25^.

Model reconstructions for 21,000, 14,000, 11,000 and 6,000 years BP (Fig. 3) further suggest that suitable climate habitat for aspen was consistently available since the last glacial maximum for aspen populations in the southwestern Rocky Mountains. These areas were not covered by continuous ice sheets and the complex landscape would have supported ample refugia for the southwestern genetic cluster of aspen populations identified by Callahan *et al*.^20^. Only minimal migration, primarily along elevational gradients, would have been required to maintain suitable habitat conditions for aspen populations in montane areas of Wyoming, Utah, Colorado, Arizona and New Mexico (Fig. 3).

### Alternate recolonisation patterns

We should note that a southwards expansion from an isolated and genetically depauperate refugial population in Alaska could also explain the observed high degree of suboptimality in the British Columbia populations. However, data reported by ref. 20 does not support a strong influence of genetic material from Beringian refugia even if such refugia existed for aspen as indicated by pollen data^34^. Our habitat reconstructions are ambivalent in this resepect, with low probabilities of presence indicated for very restricted areas in Alaska at the last glacial maximum. The southward recolonisation hypothesis (Scenario 2) seems therefore unlikely based on molecular genetic information and habitat reconstructions. Consequently, while a Beringian refugium for aspen should not be excluded, it does not appear to be the origin of today’s boreal aspen populations.

The post-glacial migration scenario (Scenario 3) with populations from three refugia making contact in Alberta could potentially explain relatively high levels of genetic diversity observed in one study in this region^12^. While we did find high levels of genetic diversity in quantitative traits in the Alberta Foothills region (Table 1), alternative explanations have previously been proposed for this observation^17, 22^. Under the more favourable environmental conditions in the foothills, sexual reproduction and successful seedling establishment is more common^6^, and becomes a driver for generating and maintaining genetic diversity through recombination.

### Stable habitat and ancient clones

The apparent continuity of suitable habitat conditions for aspen populations in montane areas of Wyoming, Utah, Colorado, Arizona and New Mexico (Fig. 3) raises the possibility that habitat conditions within the climatic tolerances of aspen were available at a single location, potentially supporting ancient clones that survived one or more glacial cycles. The largest and putatively oldest aspen clone, known as “Pando”, occupies 43 ha in the Fish Lake National Forest in south central Utah. The age of this clone has been subject to speculation that it could be several millions of years old and having survived multiple glaciations^2, 4, 5^. On the other hand, molecular studies suggest that Pando may in fact be of relatively young age^17^.

Our climate habitat reconstructions support the view that ancient aspen clones are unlikely to be found in the southwestern Rocky Mountains. While suitable climate habitat for aspen was available in the general area at all times since the last glaciation, our model hindcasts suggest that the difference between today’s climate conditions and those of the last glacial maximum were too large to stay within the climatic tolerances of aspen at any single location (and without any migration response along elevational gradients). This is illustrated in Fig. 4 at small scale for the area of the “Pando” clone, and in Fig. 5 showing the lack of overlapping habitat between 21,000 years BP and the current reference climate. Our model predicts only a few patches of stable habitat in northern Mexico and the eastern United States from which no exceptionally large clones have been documented.

It should be noted that species distribution models are generally not considered reliable enough to reconstruct species distributions at small scales for various reasons that are discussed in depth elsewhere e.g.,^45, 46^. In our reconstructions shown in Fig. 4, false positive habitat predictions would primarily be caused by not incorporating other important habitat parameters, such as soils. False negative projections would primarily be caused by the inability to model microclimate conditions that allow aspen to persist in complex terrain for both current and past climates. Both false positives and negatives would also be caused by the coarse scale of general circulation models, which prevent the reconstruction of changes to small scale weather patterns that determine local climate conditions.

Our inferences, however, do not rely on precise spatial reconstructions of past aspen distributions. Rather, Figs 4 and 5 should be more generally interpreted to imply that the magnitude of climate change between the last glacial maximum and current conditions exceeds the full climate envelope of the species’ realised niche. In the case of aspen, a pioneer species often found in marginal environments, the realised niche is likely a good proxy for the species’ climatic tolerances. Further, climatic tolerances of individual populations within the species range and of individual clones within populations will be substantially narrower than for the species as a whole. Therefore, stable habitat conditions that fall within the environmental tolerances of individual clones and allow them to persist at the same location through full glacial cycles appear unlikely for the southwestern Rocky Mountains.

## Materials and Methods

### Climate data

Climate data were generated according to Hamann, *et al*.^47^, available for anonymous download at http://tinyurl.com/ClimateNA. We use a 1961–1990 climate normal baseline dataset generated with the Parameter-elevation Regressions on Independent Slopes Model (PRISM) for monthly average minimum temperature, monthly average maximum temperature and monthly precipitation^48^. From 36 monthly variables, six biologically relevant climate variables were derived that account for most of the variance in climate data while avoiding multicollinearity: the number of growing degree days above 5 °C, mean maximum temperature of the warmest month, temperature difference between mean January and mean July temperatures, mean annual precipitation, April to September growing season precipitation, and November to February winter precipitation. The procedure of selecting these climate variables is described in more detail in Worrall, *et al*.^49^, Supplement 1. The algorithms to estimate biologically relevant variables from monthly temperature and precipitation surfaces are explained in detail by Rehfeldt^50^. To represent palaeoclimatic conditions, we overlaid the 1961–1990 baseline climate with temperature and precipitation anomalies for 6,000, 11,000, 14,000 and 21,000 years before present, generated by the Community Climate Model (CCM1) developed by the National Center for Atmospheric Research (NCAR)^51^. Subsequently the same derived variables were generated as above.

### Species distribution modelling

Past aspen habitat was reconstructed using a species distribution model for aspen based on more than 600,000 presence/absence data points from forest inventory plots, ecology plots and herbarium accessions throughout North America^49^. This model employs a regression tree ensemble classifier to relate climate variables to aspen census data, implemented by the *randomForest* package^52^ for the R programming environment^53^. Model hindcasts were validated, using the area under the curve (AUC) of the receiver operating characteristic^54^, against 9,568 records of combined fossil pollen, macrofossil, and pack rat midden (Neotoma species) data, drawn from the Neotoma Palaeoecology Database (www.neotomadb.com) for the time periods considered. Further details on fossil data sources and validation methods can be found in Roberts & Hamann^38^. To identify areas where aspen clones may have found continuously suitable habitat throughout glacial cycles, we multiplied projected aspen probability of presence layers for the 1961–1990 baseline period with projected aspen probability of presence for 21,000 years BP.

### Common garden experiments

In this paper, we also reanalyze data from a large-scale common garden trial in a different context. We use a standard statistical design widely used in provenance testing, implementing a randomised complete block (RCB) design with 43 provenances planted in 5-tree row plots, in 6 blocks, at each of 5 sites. Provenances are open-pollinated single-tree seed collections from six ecological regions in the northern portion of the species range (Fig. 1); for further details refer to^24^. The measured traits were tree height, timing of bud break and timing of leaf senescence. Tree height was measured for 6,450 trees after nine growing seasons in the field in autumn of 2006 for all five test sites. Phenological measurements, i.e. timing of bud break and timing of leaf senescence were taken on 1,290 trees at the central Alberta test site.

To visualise multi-trait genetic differentiation of the 43 seed sources, as well as the multivariate differences in the climate conditions of the seed source locations, we use principal components analysis implemented with the *FactoMineR* package^55^ for the R programming environment^53^. For the genetic ordination, traits summarised into principal components were height at five sites plus bud break and leaf abscission measured at one site (7 variables). For the climatic ordination, nine variables were used to describe the multivariate climate space more completely (Fig. 2).

### Within-population variance

The common garden trial was primarily meant as a provenance experiment to investigate genetic differentiation in adaptive traits among populations. However, it can also be used to estimate regional within-population phenotypic variation by calculating residual variance components. Since all provenances experience the same environmental conditions at a given test site, differences in the residual phenotypic variance components can be attributed to different levels of genetic variance (including dominance and epistatic genetic effects that we cannot quantify). We therefore refer to differences in the residual variance components as differences in within-population genetic variation hereafter. Strictly speaking, they are differences in within-population phenotypic variation with the environmental variance component held constant (although we cannot quantify its absolute value). To estimate variance components, we use a random-term linear model implemented with PROC MIXED of the SAS statistical software package^56^:

1 $${Y}_{ijkl}=\mu +{P}_{i}+{S}_{j}+{(P\times S)}_{ij}+B{(S)}_{(j)k}+{(P\times B(S))}_{(i(j)k)}+{e}_{l(ijk)}$$

where *Y*_*ijkl*_ is the phenotypic observation of a trait made for the *l*-th tree of a row plot, belonging to the *i*-th provenance (*P*) grown in the *j*-th test site (*S*), in the *k*-th block (*B*) within a test site. A genotype × environment effect is given by the interaction between provenance and test site (*P* × *S*) as well as provenance and block within test site (*P* × *B*(*S*)). The overall mean is indicating by *μ*, and *e*_*l*(*ijk*)_ represents the residual environmental error plus the within-family variation in each plot. The model was run separately for each region and each trait for a total of 18 model implementations. Bud break and leaf senescence were only measured at one test site (central Alberta), and in this case the test site effect does not apply and the block within site effect becomes a simple block effect. Standard errors of variance components were generated with the COVTEST option of PROC MIXED^56^.
